# Membrane Interactions and Toxicity by Misfolded Protein Oligomers

**DOI:** 10.3389/fcell.2021.642623

**Published:** 2021-03-11

**Authors:** Mario Gonzalez-Garcia, Giuliana Fusco, Alfonso De Simone

**Affiliations:** ^1^Department of Life Sciences, Imperial College London, South Kensington, United Kingdom; ^2^Department of Chemistry, Centre for Misfolding Diseases, University of Cambridge, Cambridge, United Kingdom; ^3^Department of Pharmacy, University of Naples Federico II, Naples, Italy

**Keywords:** protein misfolding, membrane interaction, receptor binding, amyloid fibrils, cellular toxicity

## Abstract

The conversion of otherwise soluble proteins into insoluble amyloid aggregates is associated with a range of neurodegenerative disorders, including Alzheimer’s and Parkinson’s diseases, as well as non-neuropathic conditions such as type II diabetes and systemic amyloidoses. It is increasingly evident that the most pernicious species among those forming during protein aggregation are small prefibrillar oligomers. In this review, we describe the recent progress in the characterization of the cellular and molecular interactions by toxic misfolded protein oligomers. A fundamental interaction by these aggregates involves biological membranes, resulting in two major model mechanisms at the onset of the cellular toxicity. These include the membrane disruption model, resulting in calcium imbalance, mitochondrial dysfunction and intracellular reactive oxygen species, and the direct interaction with membrane proteins, leading to the alteration of their native function. A key challenge remains in the characterization of transient interactions involving heterogeneous protein aggregates. Solving this task is crucial in the quest of identifying suitable therapeutic approaches to suppress the cellular toxicity in protein misfolding diseases.

## Introduction

The misfolding and aggregation of otherwise functional proteins is linked with a number of neurodegenerative disorders such as Alzheimer’s disease (AD) and Parkinson’s disease (PD), as well as non-neuropathic conditions such as type II diabetes (T2D) and systemic amyloidoses. The protein fibrils forming in association with these pathologies share common properties such as a core rich in β-sheet structure adopting a characteristic cross-β topology, the resistance to degradation and significant mechanical properties with tensile strength that is similar to steel. The precursor proteins involved in protein misfolding diseases (PMDs), however, are considerably different in their native states and range from disordered proteins (e.g., amyloid-beta – Aβ – in AD, α-synuclein – αSyn – in PD, the prion protein in prion disease) to structured monomers (e.g., beta-2 microglobulin – β2m – in β2m-amyloidosis) or globular oligomers (the transthyretin – TTR – tetramer in TTR-amyloidosis). The structural similarity between the mature amyloid aggregates has suggested the existence of some generic mechanism of toxicity. One of the recursive mechanisms includes the impairment of clearance control processes that suppress protein aggregation and toxicity in the cell, which is likely coupled with the higher prevalence of these conditions in aging populations. A detailed understanding of the molecular origins of PMDs is, however, missing, including the balance between loss of function of native proteins or gain in toxicity by aberrant aggregates.

In this review, we summarize the state of the art in the study of the mechanisms of toxicity associated with aberrant protein aggregation, with a specific focus on the interaction between protein oligomers and biological membranes, a central step at the onset and development of PMDs.

## Toxicity of Protein Oligomers

**FIGURE 1:**
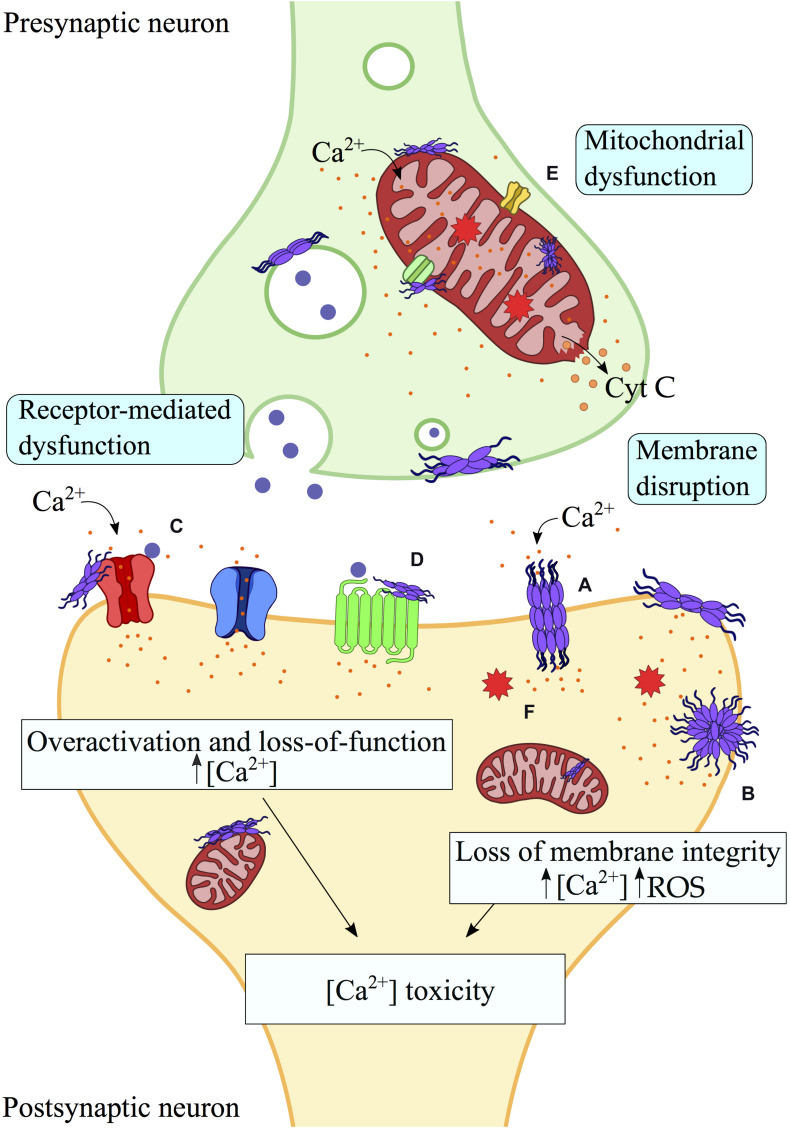
Aberrant interactions between misfolded protein aggregates and biological membranes. Toxic protein oligomers can bind strongly biological membranes, including plasma and mitochondrial membranes, as well as membrane proteins. Perturbation and pore formation **(A)** of the lipid bilayer by misfolded protein aggregates may generate membrane disruption and ultimately its permeabilization **(B)**, leading to aberrant processes such as mitochondrial dysfunction, intracellular ROS and release of cytochrome c. In addition, interactions of misfolded protein aggregates with membrane receptors **(C,D)** and mitochondrial receptors **(E)** result in loss of function (e.g., VDACs, α3-NKA) or overactivation (e.g., mGluRs, NMDARs), with consequent alteration of the cellular viability, including calcium gradient imbalance and cellular and organelle dysfunction. Indeed, most of the interactions of misfolded protein aggregates with cellular membranes and membrane proteins have a common downstream effect on the Ca^2+^-dependent toxicity. In this scheme, interactions and processes by protein oligomers at the surface of biological membranes are shown. **(A)** Pore formation. **(B)** Membrane permeabilization. **(C)** Interaction with Ionotropic/metabotropic glutamate receptors. **(D)** Interaction with plasma membrane receptors (e.g., α7 nAChR, α3-NKA, etc.). **(E)** Mitochondrial receptors interactions (VDACs, mPTP, etc.). **(F)** Intracellular ROS production. Protein aggregates are schematically shown in purple, Ca^2 +^ ions in orange and neurotransmitter molecules in blue.

It is now recognized that the most toxic species in the etiology of PMDs are the small and diffusible prefibrillar oligomers forming during protein aggregation or released by mature fibrils (Hartl, 2017). Protein oligomers have been shown to induce higher levels of cellular toxicity than mature fibrils. This has been consistently observed in many protein systems including Aβ (Walsh et al., 2002) and tau (Ward Sarah et al., 2012) in AD, αSyn (Winner et al., 2011) in PD and human islet amyloid polypeptide (IAPP) in type II diabetes (Abedini et al., 2016; Scollo and La Rosa, 2020). Compared to mature fibrils, protein oligomers have specific molecular structural characteristics that enhance their ability to induce neurotoxicity, including small size (Mannini et al., 2014), exposure of hydrophobic surfaces (Vivoli Vega et al., 2019) and unsaturated edge-strands (De Simone et al., 2008). The transient and heterogeneous nature of these species, however, makes it elusive to study their properties *in vitro* or *in vivo.* Recent investigations have been successful in identifying some of the key properties at the origin of the cellular toxicity by misfolded protein oligomers, including the disruption of the mitochondrial function (Westermark et al., 2011; Ghio et al., 2019), the induction of increased levels of basal calcium (Angelova et al., 2016), the generation of metal dyshomeostasis (Angelova et al., 2016) and stimulation of reactive oxygen species (Cheignon et al., 2018). A central role in most of these mechanisms of toxicity is the interaction between misfolded protein oligomers and biological membranes, including plasma and mitochondrial membranes (Figure 1), which will be overviewed here with respect to two major models, namely the membrane disruption and receptor-mediated interaction.

## Disruption of Biological Membranes by Toxic Protein Oligomers

**FIGURE 2:**
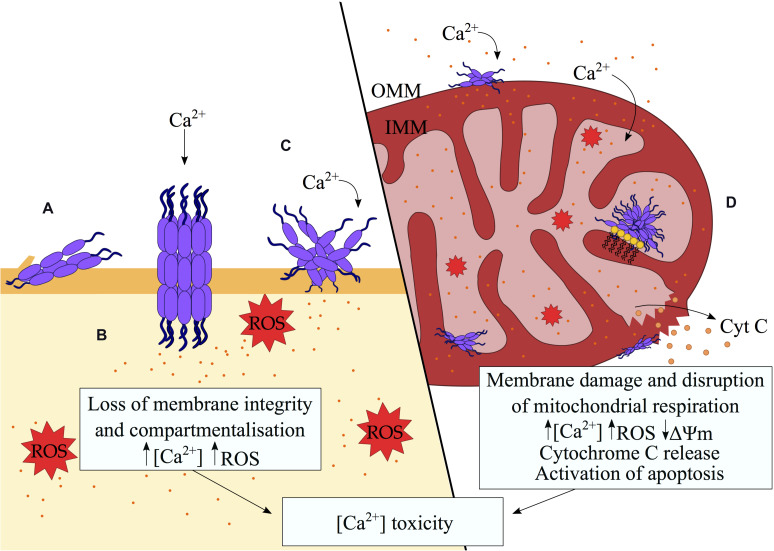
Disruption of biological membranes by misfolded protein oligomers. A variety of mechanisms of membrane disruption by misfolded protein oligomers were proposed including **(A)** partial insertion of the oligomers into the hydrophobic region of the membrane, **(B)** pore formation, **(C)** membrane thinning and redistribution of lipids through a detergent-like mechanism (Sciacca et al., 2018). The consequent loss of membrane integrity results in aberrant Ca^2+^ influx, the generation of ROS and the alteration of signaling pathways, ultimately inducing cell death in a Ca^2+^-dependent manner. **(D)** In the mitochondria, preferential binding to cardiolipin (depicted as yellow lipids) by misfolded protein oligomers promotes aberrant protein-membrane interactions. Defects in the mitochondrial membranes lead to ionic gradient imbalances, which in turn interrupt the electron transport chain and compromise the process of mitochondrial respiration. A defective mitochondrial electron transport chain, in association with elevated Ca^2+^ influx result in an increase in intracellular ROS. The loss of mitochondrial membrane potential signals the release of cytochrome c.

Oligomers in PMDs have been shown to disrupt a variety of cellular processes, including alterations of the calcium signaling (Quist et al., 2005; Kim et al., 2018) due to the disruption of the plasma membrane (Lin et al., 2001; Angelova et al., 2016). Several molecular mechanisms have been identified for the plasma membrane permeabilization by various types of misfolded protein oligomers (Figure 2). In the case of αSyn, oligomers are believed to disrupt the neuronal membrane by binding the lipid bilayer *via* the N-terminal region of the protein and by inserting their core into its hydrophobic interior (Fusco et al., 2017). This mechanism favors fluxes through the neuronal membrane of small ions and larger molecules such as the calcein dye (Cascella et al., 2019). By directly targeting the lipophilic region of the oligomers using antibodies, the toxicity of αSyn aggregates is suppressed, as shown *in vitro* and *C. elegans* (Cascella et al., 2019). The permeabilization of the plasma membrane upon interaction with protein oligomers has also been shown by electrophysiology studies of membrane conductance (Feng et al., 2010). These observations are consistent with the formation of pores of different dimensions in the membrane upon treatment with oligomers (Wong Su et al., 2018), as observed using atomic force microscopy (Chaudhary et al., 2016; Oropesa-Nuñez et al., 2016; Bode et al., 2019). The ability to generate these pores has been associated to the exposure of hydrophobic regions (Vivoli Vega et al., 2019) and the size of the protein oligomers (Mannini et al., 2014; Chiti and Dobson, 2017).

Another model is based on evidence supporting the formation of ion channels by protein oligomers at the origin of the pathophysiology for amyloid diseases. Aβ42 has been shown to form discrete pore structures ranging between 1.7 and 2.4 nm in diameter (Bode et al., 2017). The architecture and dimensions of these pores resemble annular Aβ oligomers that had previously been shown to form tetrameric and hexameric channel-like structures by atomic force microscopy (Lin et al., 2001; Quist et al., 2005; Jang et al., 2010), which were also shown to generate discrete current bursts. Similar channel-like structures have been reported for αSyn (Lashuel et al., 2002; Kayed et al., 2009; Chen et al., 2015) having ion channel-like activity (Kim et al., 2009; Tosatto et al., 2012). These findings suggest an additional common mechanism of alteration of ion homeostasis in the pathogenesis of PMDs.

The membrane interaction by misfolded protein oligomers is also strongly influenced by the lipid composition, which is a modulator of the downstream cellular toxicity. An increase in the content of gangliosides and cholesterol in the cellular membrane enhances the toxicity of misfolded oligomers of the HypF-N protein model system *in vitro* (Evangelisti et al., 2012). This is consistent with evidence of a correlation between age-related alterations of the lipid homeostasis and neurodegenerative diseases, as well as with altered fractions of gangliosides in brains of Alzheimer’s patients (Yamamoto et al., 2008), including membrane fractions from the frontal cortex and the temporal cortex (Molander-Melin et al., 2005) as well as in lipid rafts (Liu et al., 2015a).

Neurodegenerative conditions also feature lipid peroxidation due to oxidative stress, which has been shown to enhance protein misfolding and aggregation as well as to promote membrane permeability (Liu et al., 2008; Choi and Gandhi, 2018; Michalska and León, 2020). This generates a positive feedback mechanism in which the peroxidation of the membrane triggers more vigorous protein aggregation, as shown for Aβ (Pilkington et al., 2019) and αSyn (Angelova et al., 2020a), which in turn generates additional oligomers, thereby enhancing the oxidative stress and lipid peroxidation. Products of lipid peroxidation have been found in brains of both PD and AD patients, as well as in animal models of such diseases (Joshi et al., 2006; Picklo and Montine, 2007; Dalfó and Ferrer, 2008; Padurariu et al., 2010). Peroxidation leads to defects in the packing of the phospholipids and weakens cellular membranes, making them more vulnerable to pore formation induced by protein aggregates. Oxidative stress also alters the chemical integrity of proteins, which is also a way to promote aggregation of amyloidogenic proteins (Angelova et al., 2020b), leading to a feed-forward loop where aberrant interactions with membranes are intensified.

It has also been proposed that complexes between misfolded proteins and free lipids in solution would be at the origin of membrane disruption in PMDs (lipid-chaperone hypothesis). The model suggests that pre-binding to lipids would favor the insertion of oligomers into the membrane in a chaperone-like mechanism (La Rosa et al., 2016; Scollo et al., 2018; Sciacca et al., 2020).

## Mitochondrial Membrane Disruption

Oxidative phosphorylation in the mitochondria is responsible for most of the energy required for normal cellular function, a process that is particularly relevant for neuronal cells where a significant energetic consumption is required for synaptic transmission (Attwell and Laughlin, 2001). The mitochondrial dysfunction has been associated to most PMDs (Cenini et al., 2019), and evidence exists for a role of misfolded protein oligomers in the impairment of the mitochondrial function. Post-mortem brains of PD and AD patients indeed revealed the co-localization of aggregates of Aβ, αSyn and tau with mitochondrial membranes (Hsu et al., 2000) and mitochondrial-associated membranes (Guardia-Laguarta et al., 2014). *In vitro* and animal studies have shown that prefibrillar oligomers induce neuronal death following mitochondrial membrane damage and mitochondrial dysfunction (Lashuel, 2005; Glabe, 2006; Cenini et al., 2019; Farrugia et al., 2020; Figure 2). Misfolded protein oligomers from tau, Aβ, IAPP, and αSyn have generally high affinity for the lipid composition of mitochondria, and particularly for their cardiolipin component (Camilleri et al., 2013, 2020; Kegulian et al., 2015; Ghio et al., 2019). Cardiolipin is a fundamental phospholipid that is exclusively found in mitochondrial membranes, where it plays a crucial role in many biochemical processes, including respiration and signaling. The characteristic structure of cardiolipin, which is formed by two phosphatidyl residues and four acyl chains joined by a glycerol bridge, has a profound effect on the curvature and packing of mitochondrial membranes (Horvath and Daum, 2013) and promotes the binding of protein oligomers (Elías-Wolff et al., 2019).

In the pathophysiology of PD, mitochondrial dysfunction is a strong element inducing neuronal impairment. Transmission electron microscopy analysis of dopaminergic neurons overexpressing wild-type αSyn demonstrated abnormal mitochondrial morphologies, including fragmentation and malformed cristae (Ganjam et al., 2019). Indeed, several mutations in genes responsible for mitochondrial and reactive oxygen species (ROS) homeostasis (PINK1, PINK2 (Parkin), LRRK2, and DJ-1) have been shown to lead to PD (Bonifati et al., 2002; Valente et al., 2004; Gilks et al., 2005; Nichols et al., 2005). *In vitro* incubation of αSyn with isolated mitochondria (Ghio et al., 2019) results in the formation of membrane pores in a cardiolipin-dependent manner. Amounting evidence has demonstrated the accumulation of αSyn specifically on the internal mitochondrial membrane (IMM) (Devi et al., 2008), which is rich in cardiolipin (20% of total lipids) compared to the outer mitochondrial membrane [OMM (5% of lipid content)] (Klingenberg, 2009; Horvath and Daum, 2013; Pariary et al., 2019). Prefibrillar αSyn oligomers have been shown to trigger an unregulated influx of Ca^2+^, leading to mitochondrial calcium overload in isolated mitochondria and in dopaminergic neuronal models (Luth et al., 2014; Angelova et al., 2016). This induced the opening of the mitochondrial permeability transition pore (mPTP) (Luth et al., 2014), triggering the depolarization of the mitochondria (Sherer et al., 2002; Banerjee et al., 2010; Nakamura, 2013) and the release of cytochrome c (Hsu et al., 2000; Devi et al., 2008; Luth et al., 2014), leading in turn to the activation of cytoplasmic caspases signaling for a downstream apoptotic response (Ganjam et al., 2019).

Consistent with these findings, tau oligomers, but not monomers or fibrils, were able to disrupt model mitochondrial membranes in a cardiolipin-dependent manner (Camilleri et al., 2020), whereas the ability of tau to inhibit calcium efflux from the mitochondria was shown to induce mitochondrial depolarization in cortical neurons with consequent calcium-induced caspase 3 activation (Britti et al., 2020). Emerging evidence reported the perturbation of the mitochondrial membranes by Aβ42 oligomers (Glabe, 2006; Reddy, 2009; Camilleri et al., 2013; Oren et al., 2020) resulting in the impairment of calcium homeostasis, the reduction in the activity of respiratory chain complex I and IV (Mutisya et al., 1994; Casley et al., 2002; Rhein et al., 2009), and the induction of apoptotic pathways (Resende et al., 2008; Umeda et al., 2011; Bhat et al., 2015).

In the context of amyotrophic lateral sclerosis (ALS), aggregates of superoxide dismutase 1 (SOD1) have been associated with widespread mitochondrial dysfunction and mitochondrial membrane perturbation, as mitochondrial defects have been observed in tissues from ALS patients (Hirano et al., 1984). SOD1 aggregates were observed to bind the cytoplasmic leaflet of the OMM (Deng et al., 2006; Lin and Beal, 2006) and localize with mitochondria in a tissue-dependent manner (Vande Velde et al., 2008). Mitochondrial dysfunction as a result of membrane integrity defects caused by misfolded SOD1 has been reported *in vitro* in neuronal cells (Carrì et al., 1997; Menzies et al., 2002) and *in vivo* in mutant SOD1 transgenic rodent models (Higgins et al., 2002; Mattiazzi et al., 2002), as well as in ALS patients (Swerdlow et al., 1998; Wiedemann et al., 2002). Moreover, *in vitro* incubation of SOD1 oligomers and fibrils was observed to lead to the permeabilization of the membranes of isolated rat brain mitochondria (Oladzad Abbasabadi et al., 2013).

Perturbation of mitochondrial membranes is also found in non-neuropathic amyloidoses, including lysozyme systemic amyloidosis, T2D and type I diabetes (T1D) (Gurlo et al., 2010; Westermark et al., 2011, 2017). IAPP aggregates, associated with the pathology of T2D, have been shown to perturb mitochondrial membranes and accumulate at mitochondrial cristae (Kegulian et al., 2015). Membrane-associated IAPP oligomers have also been shown to have a general ability to permeate the plasma membrane through the formation of small and large pores, of which only the latter were considered cytotoxic (Pannuzzo et al., 2013; Birol et al., 2018). Indeed, mitochondrial membrane depolarization downstream of membrane damage (Birol et al., 2018) has been associated with cellular dysfunction and apoptosis in T2D (Gurlo et al., 2010). Whether disruption of the mitochondrial membranes is the initial step, or a major amplifier in the process of cell death leading to loss of β-cells in the pancreas during the development of diabetes, remains unclear. Similarly, perturbation of mitochondrial membranes by prefibrillar oligomers but not monomers or fibrils of hen egg white lysozyme has been observed *in vitro* using isolated rat brain mitochondria (Meratan et al., 2011; Meratan and Nemat-Gorgani, 2012), with membrane defects causing the release of mitochondrial enzymes and cytochrome c (Meratan et al., 2011).

## Receptor-Mediated Toxicity by Protein Oligomers

**FIGURE 3:**
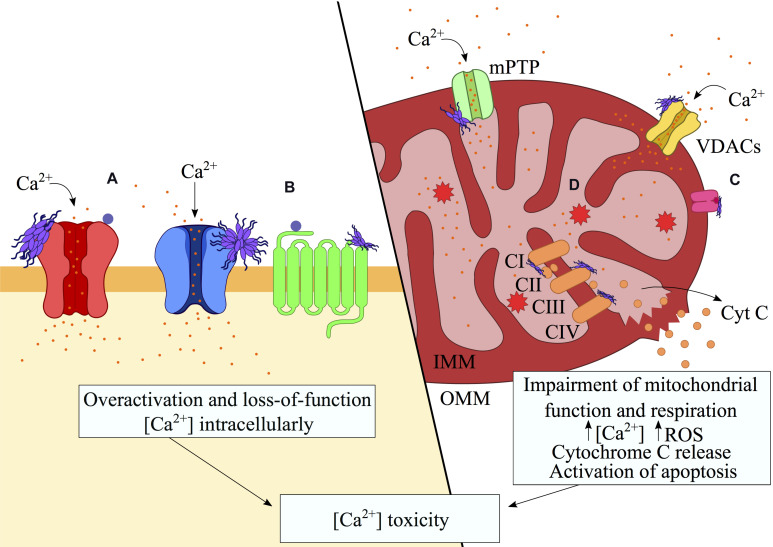
Aberrant interactions of misfolded protein oligomers with membrane receptors. Misfolded protein oligomers are able to bind a variety of proteins on the plasma and mitochondrial membranes, resulting in the alteration of their functions. These interactions may result in overactivation and hence gain-of-function (e.g., mGluR5s or NMDARs) whereas internalization (e.g., AMPARs, α7 AChR) and clustering of transporters (e.g., α3-NKA) may lead to loss-of-function. The binding to the receptors has been shown to alter the ionic gradients and generate an excess of intracellular and mitochondrial Ca^2+^. The resulting opening of the mPTP causes the depolarization of the mitochondria, which triggers the release of cytochrome c and consequent signaling for apoptosis. Disruption of the electron transport chain by direct binding of oligomers to the respiratory complexes exacerbates the calcium-mediated toxicity through the unregulated generation of ROS. In this schematic figure, interactions with **(A)** ionotropic/metabotropic glutamate receptors, **(B)** nicotinic receptors (e.g., NKA transporter) and **(C)** mitochondrial receptors (e.g., TOM20), are shown in association with **(D)** the resulting ROS generation.

In addition to promoting loss of membrane integrity, misfolded protein aggregates have been proposed to induce toxicity *via* direct interaction with membrane proteins (Figure 3). The “receptor-mediated model” of toxicity of misfolded protein oligomers involves a plethora of membrane proteins and results in the transduction of deleterious signals and ultimately cellular toxicity.

The glutamate receptor family – metabotropic glutamate receptors (mGluR), *N*-methyl-D-aspartate receptors (NMDAR) and AMPA receptors (AMPAR) – has emerged as a key target for the intracellular transduction of toxic signals by misfolded proteins. The metabotropic glutamate mGluR5 receptors are amongst the major receptors for Aβ aggregates, as their co-localization has been repeatedly reported (Casley et al., 2009; Lim et al., 2013; Shrivastava et al., 2013). Indeed, knockout of mGluR5s results in a reduction in neurodegeneration induced by Aβ oligomers (Hamilton et al., 2014). Trapping of mGluR5s receptors by Aβ aggregates results in the increased localization of the glutamate receptors at excitatory synapses and dendritic spines, generating a constitutively active signaling complex that leads to an increased mGluR5s-dependent intracellular Ca^2+^ signal (Renner et al., 2010). In the presence of Aβ oligomers, mGlu5Rs mediate the excessive release of Ca^2+^ from the endoplasmic reticulum (ER) in neurons and consequently lead to cellular toxicity (Renner et al., 2010; Um et al., 2013). Indeed, upregulation of mGlu5Rs has been observed in brains of PD patients, suggesting a putative role for mGlu5Rs receptors in protein oligomer pathology in PD (Sanchez-Pernaute et al., 2008; Price et al., 2010; Morin et al., 2013). In general, an increased expression of mGlu5Rs seen in AD, PD and other neurodegenerative diseases results in an elevated availability of mGlu5Rs at the synapses, which is likely to be responsible for the observed rise in intracellular Ca^2+^ and consequent calpain activation, ER stress and cell death (Mattson, 2007).

Oligomeric Aβ may also contribute to neurotoxicity through the overactivation of the ionotropic glutamate receptor NMDA, which is involved in synaptic plasticity stimulation, memory, learning and the assembly of neuronal connections (Li and Tsien, 2009). By interfering with the function of NMDA receptors, Aβ oligomers trigger an abnormal influx of Ca^2+^ into neurons, which can lead to excitotoxicity (Alberdi et al., 2010; Liang et al., 2017). Aβ oligomers also stimulate the internalization of AMPA receptors in a calcineurin-dependent manner (Hsieh et al., 2006), resulting in receptor depletion from the postsynaptic surface, as well as trafficking of AMPAR to the membrane upon synaptic potentiation (Rui et al., 2010). Additionally, the potential coupling of NMDA receptors with other synaptic receptors such as α3-NKA and mGlu5Rs, could compromise the physiological function of NMDA, thereby impairing long-term potentiation (LTP) (Hu et al., 2014).

The association between Aβ and the cellular prion protein (PrP^*C*^) has recently attracted a large attention. PrP^*C*^ has putatively high affinity for Aβ oligomers and has been suggested to induce neurotoxic processes through the mediation of Aβ toxicity (Laurén et al., 2009; Salazar et al., 2017; Brody and Strittmatter, 2018; Purro et al., 2018; Zhang et al., 2019), leading to the impairment of synaptic plasticity and defects in LTP in AD (Freir et al., 2011). Acting as an extracellular platform with the ability to coordinate multiprotein interactions, PrP^*C*^ has been proposed to mediate downstream signaling intracellularly from the cell surface upon Aβ binding (e.g., mGluR5, α7 AChR) (Um et al., 2013; Haas et al., 2014, 2016; Hu et al., 2014; Zhang et al., 2019). Specifically, the interaction between Aβ and PrP^*C*^ has been implicated in dendritic spine loss through the activation of the Fyn kinase, and the consequent phosphorylation of the NR2B subunit of the NMDA receptor (Um et al., 2012; Brody and Strittmatter, 2018). Downstream of Fyn activation is the tyrosine kinase Pyk, which has been shown to phosphorylate tau (Li and Götz, 2018), a step associated with AD (Iqbal et al., 2010). Furthermore, the Aβ/PrP^*C*^ interaction can recruit the low-density lipoprotein receptor-related protein-1 (LRP1) to facilitate the cell internalization of Aβ aggregates (Rushworth et al., 2013). LRP1 has also been implicated in tau endocytosis and spread (Rauch et al., 2020), and may therefore potentially control the internalization of both toxic forms of tau and Aβ in AD. More generally, PrP^*C*^ has been described as a mediator of the toxicity of a number of misfolded proteins involved in neurodegeneration. αSyn and tau have been shown to bind immobilized PrP, on mouse cortical neurons and *in vivo* (Ferreira et al., 2017; Corbett et al., 2020). In addition, the interaction between αSyn and PrP^*C*^ appears to induce aberrant Ca^2+^ levels and synaptic deficits in an mGluR5-mediated manner involving activation of the NMDA receptor (Ferreira et al., 2017).

In addition to a gain of function of membrane receptors, the interaction with protein aggregates may also result in loss of function, as in the case of the α3-Na^+^/K^+^ ATPase (α3-NKA) sodium pump upon binding with Aβ and αSyn aggregates (Ohnishi et al., 2015; Shrivastava et al., 2015). The activity of the NKA transporter is strongly associated with the brain energy consumption, leading to the possibility that its impairment may be involved in an energy crisis in the brain (Shrivastava et al., 2017). Aβ oligomers have been shown to impair the NKA catalytic activity, whilst αSyn has been shown to compromise the ability of this transporter to readily export sodium ions as a result of co-clustering around synapses. Additionally, a recent study demonstrated the ability of mutant SOD1 to interact also with α3-NKA in ALS (Ruegsegger et al., 2016).

Misfolded protein oligomers have also been observed to interact with mitochondrial membrane proteins. Voltage-dependent anion channels (VDACs) in the mitochondrial outer membrane have been reported to play a role in the toxicity associated with protein misfolding. It has been proposed that VDAC may allow the entry of αSyn into mitochondria (Lu et al., 2013), whereas other studies suggested that αSyn induces loss of function of VDAC *in vitro* in a voltage-dependent manner, leading to a reduction in the levels of ATP and ADP that compromise mitochondrial respiration (Rostovtseva et al., 2015). In the context of ALS, aggregates of mutant variants of SOD1 have been observed to accumulate on the OMM through direct interactions with VDAC1 (Israelson et al., 2010) and Bcl-2 (Pedrini et al., 2010), causing VDAC dysfunction leading to altered membrane potential, morphology and protein trafficking in mitochondria. Binding of mutant but not wild-type SOD1 to Bcl-2 induces a conformational change that uncovers its BH3 (death) domain. These processes lead to an increase in the affinity of Bcl-2 for VDAC, which in turn triggers the closure of the channel (Tan et al., 2013) and ultimately the activation of mitochondrial apoptosis. These steps appear to contribute to the initiation and development of motor neuron loss in ALS (Tafuri et al., 2015).

In the context of Huntington’s disease (HD), mutant huntingtin (Htt) has been associated with the opening of mPTP in the inner mitochondrial membrane, triggering the release of cytochrome c and activating apoptosis (Choo et al., 2004). Moreover, changes in mitochondrial membrane potential are mediated by abnormal high levels of intracellular Ca^2+^ as a result of an excessive release of calcium from the ER following the interaction between Htt and the InsP3 receptor (IP3R) as well as an increased influx upon NMDAR overstimulation (Damiano et al., 2010). The latter is mediated by polyglutamine-expanded Htt through disruption of the physiological interaction between normal Htt and PSD95, a scaffold protein associated with NMDA-receptor function (Sun et al., 2001; Song et al., 2003). The overactivation of NMDA-receptors may indeed be a key factor in the etiology of HD, in what is known as the excitotoxicity hypothesis that postulates that neuronal degeneration in HD results from a hyperactivation of glutamate receptors following an abnormal glutamatergic neurotransmission (DiFiglia, 1990). Injection of NMDA agonists into the striatum of rats induced indeed the degeneration of GABAergic neurons in a pattern reminiscent of the pathology of HD (Brouillet et al., 1999).

Alzheimer’s disease is also associated with impairment of the cholinergic pathway in the cerebral cortex and basal forebrain, which involves two main classes of receptors, muscarinic (mAChR) and nicotinic acetylcholine receptors (nAChR) (Sarter and Paolone, 2011; Maurer and Williams, 2017). A decrease in the number of nAChRs in AD results from the loss of forebrain cholinergic neurons and a cholinergic presynaptic denervation (Hampel et al., 2018). It has been postulated that under physiological conditions, Aβ42 may enhance synaptic plasticity and memory through the stimulation of α7 nAChR (Puzzo et al., 2015). Under pathological conditions, however, accumulation of aggregates of Aβ would lead to the internalization and impairment of α7 AChR in a negative feedback loop, ultimately resulting in synaptic alteration and loss of memory. Evidence suggests that the complex formed between Aβ and α7 nAChR would be able to induce tau hyperphosphorylation (Wang et al., 2003) and promote plaque formation (Nagele et al., 2003; Dineley et al., 2007), which are both hallmarks of AD. In order to compensate for the internalization of the Aβ-α7 nAChR complex, α7 nAChR is upregulated (Shen and Wu, 2015) with further internalization of the complex leading to the accumulation of Aβ, the overwhelming of the lysosomes and ultimately neuronal lysis (Palop and Mucke, 2010). In addition to an Aβ-dependent internalization of α7 nAChR, upregulation of α7 nAChR in the membrane may result in elevated Ca^2 +^ influx, further contributing to neurotoxicity (Liu et al., 2015b). Interestingly, administration of α7 nAChR antagonists to rats rescued learning deficits associated with overactivation of the receptors (Burke et al., 2014), hinting at a possible therapeutic opportunity for overstimulated α7 nAChRs in AD.

## Conclusion

In summary, there is growing evidence about the key relevance of the membrane interaction by misfolded protein oligomers in PMDs. Two principal mechanisms, namely the membrane disruption and the receptor binding, are supported by extensive experimental evidence. It remains, however, difficult to identify the dominant mechanism of toxicity in PMDs, primarily owing to the heterogeneous and transient nature of misfolded protein oligomers. These species have the ability to interact with a variety of cellular membranes and membrane proteins, with affinities that can be modulated by several factors, including the properties of the membrane (Chaudhary et al., 2016; Oropesa-Nuñez et al., 2016; Bode et al., 2019) and the lipid composition (Evangelisti et al., 2012; Sciacca et al., 2020). The level of biological complexity associated with these processes, therefore, poses tremendous challenges in the quest of identifying effective therapeutic strategies to suppress the cellular toxicity in PMDs, as these conditions are associated with various imbalances in cellular homeostasis, and their initiation or propagation can be triggered by multiple concurring factors.

## Author Contributions

MG-G, GF, and AD contributed to drafting different parts of the manuscript. All authors revised the manuscript critically for important intellectual content and approved the final version.

## Conflict of Interest

The authors declare that the research was conducted in the absence of any commercial or financial relationships that could be construed as a potential conflict of interest.
